# Risk Evaluation of Point-of-Care Testing (POCT) Devices: Insights From a Tertiary Care Hospital

**DOI:** 10.7759/cureus.80499

**Published:** 2025-03-12

**Authors:** Subodh S Satheesh, Pramudha Shree Mourya GV

**Affiliations:** 1 Pharmacy, Institute of Health Management Research, Bengaluru, IND; 2 Hospital Management, Institute of Health Management Research, Bengaluru, IND

**Keywords:** fmea, point of care devices, quality control testing, risk assessment, rpn

## Abstract

Introduction

Point-of-care testing (POCT) entails conducting a test near a patient, delivering rapid results outside the traditional laboratory setting. Even though point-of-care testing offers the advantage of rapid test results and enables quicker medical decisions, it also introduces unique risks of errors, raising concerns regarding the quality and reliability of these results. This study focuses on a comprehensive risk assessment of POCT devices in a tertiary care setting to evaluate performance, identify potential errors, categorize risks, and implement strategies to enhance their safety and effectiveness.

Method

The study was conducted as a prospective, observational, nonrandomized study in a tertiary care hospital. A total of 53 devices were selected purposively which were used across various departments of the hospital, including emergency, intensive care unit (ICU), critical care unit (CCU), operation theatre (OT), maternity, and obstetrics. The assessment covered a range of POCT devices: blood gas analyzers, cardiac biomarkers, coagulation analyzers, and glucometers. In POCT devices, risk assessment identifies potential hazards that may arise throughout the entire testing process, encompassing the pre-analytical, analytical, and post-analytical phases. Performance of the devices was evaluated using comparison with standard guidelines and errors were identified using direct observations and incident reports. The Failure Modes and Effect Analysis (FMEA) template served as a tool for on-site risk assessment, facilitating the systematic identification and evaluation of potential failure modes and their corresponding effects. A comparison of the scored risk priority number (RPN) before and after interventions was conducted to assess the effectiveness of the implemented suggestions or action plans for mitigating the identified risks.

Results

Inaccurate patient identification, clotted samples due to improper mixing, patient injury, and delays in sample transportation were the key risks identified during the pre-analytical phase. Delays in sample processing, improper handling of internal quality control materials, improper handling of external quality assessment scheme samples, failure to adhere to original equipment manufacturer instrument maintenance protocols, mishandling of patient samples, and inadequate quality checks were a few observations during the analytical phase. Reporting errors, delayed turnaround times (TAT), and delays in reporting panic values were observed during the post-analytical phase. Implementing quality control measures, real-time monitoring, automation, comprehensive staff training, and risk mitigation strategies served to enhance the reliability and efficiency of POCT devices.

Conclusion

In point-of-care testing devices, risk assessment identifies potential hazards that may arise throughout the entire testing process, encompassing the pre-analytical, analytical, and post-analytical phases. Continuous monitoring and improvement efforts are essential for adapting to evolving risks in POCT devices.

## Introduction

Point-of-care testing (POCT) is a rapidly evolving discipline within laboratory medicine, expanding in both analytical scope and clinical application [1]. POCT entails conducting a test near a patient, delivering rapid results outside the traditional laboratory setting. The adoption of POCT in low- and middle-income countries (LMICs) has predominantly focused on infectious diseases, including malaria, human immunodeficiency virus (HIV), human papillomavirus (HPV), dengue, Ebola and Zika viruses, as well as tuberculosis [2]. Even though POCT offers the advantage of rapid test results and enables quicker medical decisions, it also introduces unique risks of errors, raising concerns regarding the quality and reliability of these results. Unlike the central laboratory, where errors typically occur in the pre- and post-analytic phases, POCT errors are predominantly associated with the analytic phase of testing. Most issues encountered with POCT stem from unacceptable performances in internal quality control (IQC), performance-wise concerns in external quality assessments (EQAs), misinterpretation of results, calibration drift, and inadequate quality control measures [3-5]. These factors can contribute to inaccurate and unreliable test outcomes, posing significant implications for patient care.

Risk can be defined as the probability of experiencing harm or loss, and it can be assessed by considering both the likelihood of harm occurring and the severity of its consequences. Risk analysis encompasses several sequential steps, including risk assessment, risk communication, and risk management [6]. Risk assessment is a structured procedure to identify, analyze, and mitigate hazards and risks inherent in a particular environment, situation, or process. It serves as a methodical decision-making tool to determine appropriate measures for eliminating or controlling identified risks.

Risk management is a method by which we can reduce errors with POCT. In POCT devices, risk assessment identifies potential hazards that may arise throughout the entire testing process, encompassing the pre-analytical, analytical, and post-analytical phases [7]. This includes the initial order placement through sample collection, sample transport, processing, analysis, result reporting, and communication of results. This study aims to conduct a comprehensive risk assessment of POCT devices in a tertiary care setting. The study focuses on assessing device performance, understanding potential sources of error, categorizing identified risks, and recommending strategies to improve the safety and effectiveness of POCT devices.

## Materials and methods

The study was conducted as a prospective, observational, non-randomized study in a tertiary care hospital. A total of 53 devices were selected purposively and it focused on evaluating POCT device usage across various departments of the hospital, including emergency, intensive care unit (ICU), critical care unit (CCU), operation theatre (OT), maternity, and obstetrics. The study was carried out for a period of four months and a range of POCT devices were selected based on their high utilization and their significance in patient care. 

The devices evaluated in the study include blood gas analyzers, cardiac biomarkers, coagulation analyzers, and glucometers in the emergency department; blood gas analyzers, coagulation analyzers, and hemoglobin analyzers in the OT; glucometers in the ICU and CCU; and glucometers along with urine pregnancy testing (UPT) kits in the maternity department.

The stakeholders involved in this study had at least six months of experience in POCT usage which comprised laboratory staff, clinicians, biomedical engineers, quality managers, and technical personnel responsible for handling the POCT devices. Individuals lacking experience in managing POCT devices were excluded from the study.

Data collection methods included documentation review and on-site observations. Relevant documents such as operating manuals, maintenance records, device monitoring records, and incident reports pertaining to POCT devices were scrutinized. Additionally, direct observations were made regarding POCT device usage and practices in the specified hospital departments. The study involved only technical evaluation of POCT devices so ethical approval was not required as per institutional guidelines.

The Failure Modes and Effect Analysis (FMEA) template served as a tool for on-site risk assessment, facilitating the systematic identification and evaluation of potential failure modes and their corresponding effects [8]. It often employs three primary approaches: risk priority number (RPN), action priority (AP), and criticality. Among these, RPN stands out as a numerical method providing an intuitive means of assessing risk, where a higher RPN value indicates a higher level of risk. RPN is computed based on three key factors: severity, occurrence, and detection. Severity denotes the seriousness of the problem if it occurs, emphasizing its consequences, with higher scores indicating greater severity. Occurrence reflects the likelihood of the issue transpiring, considering all potential causes and their probability of occurrence; a higher score signifies a higher probability of occurrence. Detection evaluates how easily the problem can be identified, with a higher rating indicating a lower likelihood of detection. The RPN is calculated by multiplying the scores of severities, occurrence, and detection. Each factor is typically rated on a scale of 1 to 5 to facilitate the assessment process [9] (Tables 1-3). A risk matrix approach was used in this study and the errors were categorized based on criticality. Based on the identified risks, initiatives were taken to address the same.

**Table 1:** RPN determined on severity of risk RPN: risk priority number

Severity grade	Rating	Description of severity
Catastrophic	5	Results in death/failure to make interventions
Critical	4	Results in permanent injury of life-threatening injury/delay in intervention for critical patients
Serious	3	Results in injury or impairment requiring
Minor	2	Results in temporary injury or impairment not requiring professional medical intervention
Negligible	1	Inconvenience or temporary discomfort

**Table 2:** RPN determined on detectability of risk RPN: risk priority number

Probability of detection of risk	Rating	Description
Very low	5	Control is ineffective
Low	4	Control is less likely to detect failure
Intermediate	3	Control may or may not detect the failure
High	2	Control almost always detects the failure
Very high	1	Control can detect failure

**Table 3:** RPN determined on Occurrence of risk RPN: risk priority number

Occurrence	Rating	Description of occurrence
Frequent	5	More than once in a week
Probable	4	Once every few months
Occasional	3	Once in a year
Remote	2	Once every few years
Improbable	1	Unlikely to ever happen

## Results

A total of 53 devices were selected in the study across different departments of the hospital (Table 4). A standard checklist was developed based on the guidelines from the College of American Pathologists [3]. The risk assessment of different phases was conducted to identify and rectify potential errors. Data was collected through documentation review and on-site observations. Observations were documented by the healthcare propviders and incident reports were reviewed to assess errors related to selected devices.

**Table 4:** POCT devices and risks identified

Device	Specific risks
Electrolyte analyzer	Hemolysis, calibration drift
Hemoglobin analyzer	Sampling errors, contamination
Blood glucose analyzer	Calibration errors, storage issues with strips
Blood gas analyzer	Reagent issues, storage issues with strips
Cardiac biomarkers	Reagent issues, misinterpretation of results
Coagulation analyzers	Storage issues with strips and reagents
Urine pregnancy test	Sample collection issues and expired kits

A review of the operating manual revealed discrepancies between the recommended guidelines and actual practices, particularly regarding the frequency of IQCs, reagent storage conditions, and adherence to OEM protocols, maintenance schedules, and documentation practices.

Risk assessment was done using FMEA and RPN was used for categorization of received responses. In the FMEA analysis, it is important to note that the severity of any risk typically remains constant even after interventions are implemented. Therefore, the results of the study focused on the calculation of RPN based on the severity, occurrence, and detectability of the identified risks. A comparison of the scored RPN before and after interventions was conducted to assess the effectiveness of the implemented suggestions or action plans for mitigating the identified risks. This pre- and post-intervention comparison served as the basis for concluding the study and determining the observed effectiveness of the interventions in managing the identified risks.

Pre-analytical phase

In the pre-analytical phase of sample collection, several risks were identified, including incorrect patient identification, clotted samples due to improper mixing, needle prick injuries, and delays in sample transportation (Table 5). Continuous training programs were conducted for nursing staff and phlebotomists to enhance their skills and awareness regarding proper patient identification and sample collection techniques, thereby reducing the likelihood of errors such as wrong patient identification and clotted samples. Additionally, staff members were sensitized to the critical importance of promptly transporting samples to the laboratory to prevent delays in processing and analysis. From pre- to post-data, the decrease in RPN scores shows improvement in reducing the risks.

**Table 5:** Pre- and post-implementation RPN status of pre-analytical phase risks RPN: risk priority number

Identified risk	Pre-implementation RPN	Post-implementation RPN
Wrong patient identification	24	12
Clotted sample	15	9
Injury to patient	6	0
Delay in sample transportation	12	0

Analytical phase

In the analytical phase of sample collection, various risks were identified, including the interchange of samples, improper mixing before testing, delays in sample processing, improper handling of IQC materials including irregular IQC testing and improper storage, improper handling of external quality assessment (EQA) scheme samples like poor sample handling and documentation, failure to adhere to original equipment manufacturer (OEM) instrument maintenance protocols, mishandling of patient samples, and inadequate quality checks (QC) (Table 6). Measures such as implementing strict sample labeling and tracking protocols to prevent sample interchange, enhancing staff training on proper sample handling and mixing techniques, optimizing sample processing workflows to minimize delays, ensuring proper storage and handling of IQC and EQA scheme materials, adhering to OEM instrument maintenance schedules, and reinforcing rigorous quality control measures throughout the analytical phase aimed to mitigate the identified risks and improve the overall integrity and accuracy of laboratory testing processes. The decrease in RPN score in the evaluated components shows the effectiveness of strategies implemented to mitigate the risks. In many cases, the RPN score had dropped to 0 indicating compliance with the adopted strategies.

**Table 6:** Pre- and post-implementation RPN status for analytical phase risks RPN: risk priority number; IQC: internal quality control; EQA: external quality assessment; OEM: original equipment manufacturer

Identified risk	Pre-implementation RPN	Post-implementation RPN
Interchange of sample	8	2
Improper mixing before testing	6	0
Improper handling of IQC materials	8	0
Improper handling of EQA samples	6	0
Failure to follow OEM instrument maintenance	9	2
Improper quality check	12	4

Post-analytical phase

In the post-analytical phase of sample collection, several risks were identified, including reporting errors, and delays in reporting panic values (Table 7). These risks have the potential to impact the timely and accurate communication of test results to healthcare providers and patients, thereby affecting clinical decision-making and patient care outcomes. Measures such as implementing robust quality assurance protocols for result reporting, conducting TAT analysis, enhancing communication channels between laboratory staff and clinicians to expedite result delivery, and establishing clear escalation procedures for reporting critical or panic values promptly. The effectiveness of implemented strategies is evident in the drop in RPN scores post-implementation. 

**Table 7:** Pre- and post-implementation RPN status for post-analytical phase and storage phase risks

Post analytical phase		
Identified Risk	Pre-implementation RPN	Post-implementation RPN
Reporting error	8	0
Delayed TAT	4	0
Delay in Panic value reporting	5	0
Storage-related RPN status		
Sudden surge of patient load leading to stock out	4	0
Reagent degradation	3	0
Patient sample storage not as per policy	8	2
Delayed supplies / supply stock out	4	0
Inadequate monitoring of stock records	8	3

Storage-related risks

In the category of POCT storage-related risks, several potential issues were identified. These include the sudden surge of patient load leading to stockouts, which could result in insufficient supplies to meet testing demands (Table 7). Another risk is reagent degradation due to improper storage conditions, potentially compromising the accuracy of test results. 

Additionally, deviations from the prescribed policies for patient sample storage may occur, increasing the risk of sample contamination or misidentification. Delays or stockouts in the supply chain could also disrupt testing operations. Furthermore, inadequate monitoring of stock records poses a risk of inaccuracies in inventory management, potentially leading to understocking or overstocking of supplies. Proper storage protocols and inventory management procedures were recommended to improve the storage-related concerns.

## Discussion

In our study, numerous challenges and risks were identified in the utilization of point-of-care testing (POCT) devices across various stages, including pre-analytical, analytical, and post-analytical phases. These challenges encompassed a range of issues that could potentially impact the accuracy, efficiency, and reliability of POCT results. To address these challenges effectively, our study devised strategies tailored to each stage of the POCT process. These strategies were aimed at mitigating the identified risks and enhancing the overall performance and quality of POCT services.

In the pre-analytical stage, major challenges included wrong patient identification, clotted samples due to improper mixing, patient injury, and delays in sample transportation. Sample handling issues, transportation-related concerns, and degradation of samples can impact diagnostic accuracy, thereby compromising the efficiency of the results. Studies by Iqbal et al. and Alavi et al. also had similar observations on pre-analytical errors in the hematology laboratory [9,10]. 

In the analytical phase of sample collection, risks identified were the interchange of samples, improper mixing before testing, delays in sample processing, improper handling of IQC materials and EQAS samples, failure to adhere to OEM instrument maintenance protocols, mishandling of patient samples, and inadequate quality checks (QC). A previous study by Sturgeon et al. found similar errors in immunoassay-based analytical studies [11]. 

In the post-analytical phase of sample collection, several risks including reporting errors, delayed turnaround time (TAT), and delays in reporting panic values were identified. Similar observations were found in studies by Lenicek Krleza et al. and Zemlin et al. [12,13]. To address these concerns, targeted implementation plans were developed. 

In storage-related risks, several potential issues were identified. These include the sudden surge of patient load leading to stockouts, which could result in insufficient supplies to meet testing demands, reagent degradation due to improper storage conditions, deviations from the prescribed policies for patient sample storage, and delays or stockouts in the supply chain could also disrupt testing operations; similar issues were observed in a study by Kuupiel et al [14]. 

Real-time monitoring, streamlined workflows, comprehensive training, and automation in POCT have resulted in a substantial reduction in risks associated with POCT devices. The strategic interventions adopted were instrumental in mitigating risks and ensuring high reproducibility and reliability of POCT results.

The study has given valuable insights into the risks associated with POCT devices. It has systematically evaluated the devices in different stages, identified potential challenges, and implemented measures for improvement. There were a few limitations in the study that need to be acknowledged. A limited number of POCT devices were selected for assessment in the study; a broader selection of devices could have provided a comprehensive analysis of devices. The study was conducted in a single tertiary care hospital; a multi-center approach would have enhanced the robustness and applicability of the findings. 

## Conclusions

In POCT devices, risk assessment identifies potential hazards that may arise throughout the entire testing process, encompassing the pre-analytical, analytical, and post-analytical phases. Lack of staff awareness, training, poor adherence to quality control measures, and environmental monitoring were identified as major risk factors. Action plans included comprehensive staff training, performance monitoring, and the implementation of standardized protocols and stringent quality control initiatives. These strategies align with established standards and emphasize the importance of patient safety and regulatory compliance. Furthermore, it was instrumental in improving the reliability and effectiveness of POCT devices in our institution. Strengthening POCT services is pivotal to greater efficiency and accuracy of diagnostic services, ultimately resulting in improved clinical outcomes.
